# Automated detection of brain atrophy patterns based on MRI for the prediction of Alzheimer's disease

**DOI:** 10.1016/j.neuroimage.2009.11.046

**Published:** 2010-03

**Authors:** Claudia Plant, Stefan J. Teipel, Annahita Oswald, Christian Böhm, Thomas Meindl, Janaina Mourao-Miranda, Arun W. Bokde, Harald Hampel, Michael Ewers

**Affiliations:** a Department of Neuroradiology, Technische Universität München, Munich, Germany; b Department of Psychiatry, University of Rostock, Germany; c Department for Computer Science, University of Munich, Munich Germany; d Department of Psychiatry, Ludwig-Maximilian University, Munich, Germany; e Institute for Clinical Radiology,Department of MRI, Ziemssenstrasse 1, 80336 Munich, Germany; f Centre for Computational Statistics and Machine Learning, University College London, London, UK; g Centre for Neuroimaging Sciences, King's College London, London, UK; h Trinity College Dublin, School of Medicine, Discipline of Psychiatry & Trinity College Institute of neuroscience (TCIN) & The Adelaide and Meath Hospital Incorporating The National Children's Hospital (AMiNCH), Dublin, Ireland; i Deutsches Zentrum für Neurodegenerative Erkrankungen (DZNE), Germany

## Abstract

Subjects with mild cognitive impairment (MCI) have an increased risk to develop Alzheimer's disease (AD). Voxel-based MRI studies have demonstrated that widely distributed cortical and subcortical brain areas show atrophic changes in MCI, preceding the onset of AD-type dementia. Here we developed a novel data mining framework in combination with three different classifiers including support vector machine (SVM), Bayes statistics, and voting feature intervals (VFI) to derive a quantitative index of pattern matching for the prediction of the conversion from MCI to AD. MRI was collected in 32 AD patients, 24 MCI subjects and 18 healthy controls (HC). Nine out of 24 MCI subjects converted to AD after an average follow-up interval of 2.5 years. Using feature selection algorithms, brain regions showing the highest accuracy for the discrimination between AD and HC were identified, reaching a classification accuracy of up to 92%. The extracted AD clusters were used as a search region to extract those brain areas that are predictive of conversion to AD within MCI subjects. The most predictive brain areas included the anterior cingulate gyrus and orbitofrontal cortex. The best prediction accuracy, which was cross-validated via train-and-test, was 75% for the prediction of the conversion from MCI to AD. The present results suggest that novel multivariate methods of pattern matching reach a clinically relevant accuracy for the a priori prediction of the progression from MCI to AD.

## Introduction

Alzheimer's disease (AD) is the most frequent cause of age-related dementia. Due to the increasing proportion of elderly people in the Western societies, the prevalence of dementia is projected to double within the next three decades (Ferri et al., 2005). The reliable and early detection of AD in predementia stages such as mild cognitive impairment (MCI) is the basis for the development of preventive treatment approaches. However, especially the diagnosis of mild AD and prediction of development of AD in at-risk groups remains challenging. In addition to cerebrospinal fluid derived biomarkers (Blennow and Hampel, 2003; Ewers et al., 2007; Hansson et al., 2006; Herukka et al., 2007; Zhong et al., 2007), neuroimaging markers have been recommended to be included in the revised NINCDS-ADRDA diagnostic standard criteria (Dubois et al., 2007) and proposed as predictors of AD (Winblad et al., 2004; Petersen et al., 2001). The best established MRI derived marker of AD, hippocampus volume, shows relatively high diagnostic accuracy for AD but clinically insufficient predictive value for the prediction of progression from MCI to AD when assessed as the sole predictor (Csernansky et al., 2005; Jack et al., 1999; Kantarci and Jack, 2003; Killiany et al., 2002; Pennanen et al., 2004; Stoub et al., 2005; Visser et al., 1999).

As an alternative to ROI based volumetry, automated morphometry and deformation-based approaches have been developed to map the pattern of structural brain changes across the entire brain (Ashburner and Friston, 2000; Good et al., 2001). A series of voxel-based morphometric studies in MCI and mild AD have shown marked volume differences not only within the hippocampus area but also distributed within cortical brain areas such as the precuneus and cingulate gyrus (Baron et al., 2001; Chetelat et al., 2002, 2005; Frisoni et al., 2002; Karas et al., 2004; Pennanen et al., 2005). However, few statistical approaches have been proposed to derive individual risk scores from such maps of atrophy for the clinical prediction of AD. Data mining approaches and pattern recognition methods provide a way to extract from millions of voxels within an MRI the minimal set of voxel values necessary to attain a sufficiently high accuracy for the prediction and diagnosis of AD. Multivariate approaches such as principal component analysis (PCA) (Friston et al., 1996), independent component analysis (McKeown et al., 1998), structural equation modeling (McIntosh et al., 1994), and support vector machine (Mourao-Miranda et al., 2005, 2006) are potential candidates but have mostly been applied to functional neuroimaging data so far. Recently, such multivariate methods have been adopted for the analysis of structural MRI to detect spatial patterns of atrophy in AD (Chen and Herskovits, 2006; Davatzikos et al., 2008; DeCarli et al., 1995; Duchesne et al., 2008a,b, 2009; Fan et al., 2008; Kloeppel et al., 2008; Misra et al., 2009; Teipel et al., 2007a; Vemuri et al., 2008). These techniques allow for deriving a single value representing the degree to which a disease-specific spatial pattern of atrophy is present in a single individual. The application of such classifiers of spatial pattern of atrophy in MCI has shown promising results for the prediction of AD (Davatzikos et al., 2008; Teipel et al., 2007a).

In the present study we applied a novel two-step approach combining a distribution free feature selection algorithm at the first stage and, at the second stage, different multivariate classifiers for case-by-case decision making. The major aims of the current study were, first, to develop a novel feature selection method to circumvent potential problems of previous approaches for feature selection including lack of statistical power due to multiple testing (Fan et al., 2008) or purely-data driven correlational patterns in unsupervised dimensionality reduction (e.g. PCA (Teipel et al., 2007a,b)). Secondly, we compared different cross-validated classifiers including support vector machine (SVM), a Bayesian classifier, and voting feature intervals (VFI) combined with unsupervised clustering algorithms to derive the minimal set of voxels for optimized prediction of diagnosis (AD vs. HC) or prediction of AD in MCI. The overall goal was to derive an optimized classification that is sensitive for the early MRI-based detection of AD.

## Materials and methods

### Subjects

32 patients with clinically probable AD, 24 patients with amnestic MCI and 18 healthy control subjects (HC) underwent MRI and clinical examinations (Table 1).

AD patients fulfilled the criteria of the National Institute of Neurological Communicative Disorders and Stroke and the Alzheimer Disease and Related Disorders Association (NINCDS-ADRDA) criteria for clinically probable AD (McKhann et al., 1984). MCI subjects fulfilled the Mayo criteria for amnestic MCI (Petersen et al., 2001). All MCI subjects had subjective memory complaints, a delayed verbal recall score at least 1.5 standard deviations below the respective age norm, normal general cognitive function, and normal activities of daily living. Severity of cognitive impairment was assessed by the Mini-Mental-State-Examination (MMSE) (Folstein et al., 1975). Controls did not have cognitive complaints and scored within 1 standard deviation from the age adjusted norm on all subtests of the CERAD cognitive battery (Morris et al., 1989).

MCI patients received clinical follow-up examinations over approximately 2.5 years, using clinical examination and neuropsychological testing to determine which subjects converted to AD and which remained stable. All subjects were only examined if they gave their written informed consent. The study was approved by the institutional review board of the Clinic of Psychiatry at the Ludwig Maximilian University of Munich.

### MRI acquisition

MRI examinations of the brain were performed on a 1.5 T MRI scanner (Magnetom Vision, Siemens Medical Solutions, Erlangen, Germany). We acquired a high-resolution T1-weighted Magnetisation Prepared Rapidly Acquired Gradient echo (MPRAGE) 3D-sequence with a resolution of 0.55 by 0.55 by 1.1 mm^3^, TE = 3.9 ms, TI = 800 ms, and TR = 1,570 ms. The FOV was 240 mm and the pixel matrix was 512 × 512.

### MRI processing

The preprocessing of the scans was conducted with the statistical software package SPM2 (Wellcome Trust Centre for Neuroimaging, London, http://www.fil.ion.ucl.ac.uk/spm/). The high dimensional normalization of the MRI scans was processed according to a protocol that has been described in detail previously (Teipel et al., 2007a,b). First, we constructed a customized template across groups averaged across images that were normalized to the standard MNI T1 MRI template, using the low-dimensional transformation algorithm implemented in SPM2 (Ashburner and Friston, 2000; Ashburner et al., 1997). Next, one good quality MRI scan of a healthy control subject was normalized to this anatomical average image using high-dimensional normalization with symmetric priors (Ashburner et al., 1999) resulting in a pre-template image. Finally, the MRI scan in native space of the same subject was normalized to this pre-template image using high-dimensional normalization. The resulting volume in standard space served as the anatomical template for subsequent normalizations of the remaining scans. The individual anatomical scans in standard space (after low-dimensional normalization) were normalized to the anatomical template using high-dimensional image warping (Ashburner et al., 1999). These normalized images were resliced to a final isotropic voxel size of 1.0 mm^3^. Finally, we derived Jacobian determinant maps from the voxel-based transformation tensors. Values above 1 represent an expansion of the voxel, values below 1 a contraction of the voxel from the template to the reference brain. The resulting Jacobian determinant maps were masked for brain matter and cerebrospinal fluid (CSF) spaces using masks from the segmented template MRI (Ashburner and Friston, 1997). To obtain the brain mask, the template brain scan was segmented into grey and white matter and CSF spaces. The grey matter (GM) and white matter (WM) compartments then were combined to obtain a brain mask excluding CSF: (GM + WM)./(WM + GM + CSF).⁎BRAIN with grey matter, white matter, and CSF representing the grey and white matter and CSF probabilistic maps obtained through segmentation and BRAIN representing the brain mask obtained from the brain extraction step in SPM2.

We took the logarithm of the masked maps of the Jacobian determinants (Scahill et al., 2002) and then applied a 10-mm full width at half maximum isotropic Gaussian kernel. The masked smoothed Jacobian determinant maps were scaled to the same mean value and standard deviation using a voxel-wise *z*-transformation:

$$z_{\text{i,k}}=\frac{x_{\text{i,k}}-{\overset{―}{x}}_{\text{k}}}{s_{\text{k}}}$$

where *x*_i,k_ is the FA value of voxel i in scan k, *x*__k_ is mean value across all *x*_i_ of scan k and s is the standard-deviation across all *x*_i_ of scan k.

### Data mining

We applied a multi-step data mining procedure including feature selection, clustering and classification to identify the best discriminating regions in brain images.

Notations Given a data set *DS* consisting of MRI scans of *n* subjects *s*_1_, …, *s*_*n*_ labeled to a set of *k* discrete classes *C *= {*c*_1_, …, *c*_k_} (in our study e.g. HC and AD), we denote the class label of subject *s*_*i*_ by *s_i_.c*. For each subject we have an MR image which is represented as a feature vector *V* composed of *d* voxels *v_1_, …, v_d_*.

#### Feature selection

First we select the most discriminating features using a feature selection criterion. We use the Information Gain (Quinlan, 1993; Hall and Holmes, 2003) to rate the interestingness of a voxel for class separation, which requires the following definitions.

Entropy of the class distribution. The entropy of the class distribution *H(C)* is defined as $H\left(C\right)=\sum_{c_{i}\in C}p\left(c_{i}\right)\cdot \log_{2}\left(p\left(c_{i}\right)\right)$, where *p(c_i_)* denotes the probability of class *c*_*i*_, i.e*.* |{*s*|*s* ∈ *DS∧ s.c = c*_*i*_}|/*n*. *H(C)* corresponds to the required amount of bits to tell the class of an unknown subject and scales between 0 and 1. In the case of *k = 2*, (e.g. we consider the two classes HC and AD), if the number of subjects per class is equal for both classes, *H*(*C*) = 1. In the case of unbalanced class sizes the entropy of the class distribution is smaller than one and approaches zero if there are much more instances of one class than of the other class.

Information Gain of a voxel. Now we can define the Information Gain of a voxel *v*_i_ as the amount by which *H(C)* decreases through the additional information provided by *v*_i_ on the class, which is described by the conditional entropy *H(C*|*v*_i_*)*.

$$IG\left(v_{i}\right)\text{=}H\left(C\right)\text{−}H\left(C\text{|}v_{i}\right).$$

In the case of *k = 2*, the Information Gain scales between 0 and 1, where 0 means that the corresponding voxel provides no information on class label of the subjects. An Information Gain of 1 means that the class labels of all subjects can be derived from the corresponding voxel without any errors.

To compute the conditional entropy, features with continuous values, as in our case, need to be discretized using the algorithm of Fayyad and Irani (1993). This method aims at dividing the attribute range into class pure intervals. The cut points are determined by the Information Gain of the split. Since a higher number of cut points always implies higher class purity but may lead to over fitting, an information-theoretic criterion based on the Minimum Description Length principle is used to determine the optimal number and location of the cut points.

#### Clustering

After feature selection, we apply a clustering algorithm to identify groups of adjacent voxels with a high discriminatory power and to remove noise. Clustering algorithms aim at deriving a partitioning of a data set into groups (clusters) such that similar objects are grouped together. We apply clustering to group voxels with similar spatial location in the brain and similar (high) IG. The density-based clustering algorithm DBSCAN (Ester et al., 1996) has been designed to find clusters of arbitrary shape in databases with noise. In our context, clusters are connected areas of voxels having a high IG which are separated by areas of voxels of lower IG. DBSCAN has been originally designed for clustering data objects represented by feature vectors. We first briefly introduce the general definitions of DBSCAN and then elaborate on the required modifications for clustering voxels. DBSCAN employs a density threshold for clustering, which is expressed by two parameters, *ɛ* specifying a volume and *MinPts* denoting a minimum number of objects. Formally, the density-based clustering notion of DBSCAN is defined as follows:

Definitions of DBSCAN An object *O* is called *core object* if it has at least *MinPts* objects in its *ɛ* range, i.e*.* |*N_ɛ_(O)> = MinPts*|, where *N_ɛ_(O) = {O′*| *dist(O, O′)< = ɛ}*. An object *O* is directly density-reachable from another object *P* with respect to *ɛ* and *MinPts* if *P* is a core object and $O\in N_{ɛ}(P)$. An object *O* is *density-reachable* from an object *P* with respect to *ɛ* and *MinPts* if there exists a sequence of objects *O_1_,…, O_n_* such that *O*_*1*_ = *P* and *O*_*n*_ = *O* and *O*_*i*__ + 1_ is directly density-reachable with respect to *ɛ* and *MinPts* from *O*_*i*_ for *1< = i< = n* . Two objects *O* and *P* are *density-connected* with respect to *ɛ* and *MinPts* if there exists an object *Q* such that both *O* and *P* are density-reachable from *Q*. A *density-based cluster* is the maximum set of density-connected objects, i.e. the transitive closure of the density reachability relation.

**Fig. 1:**
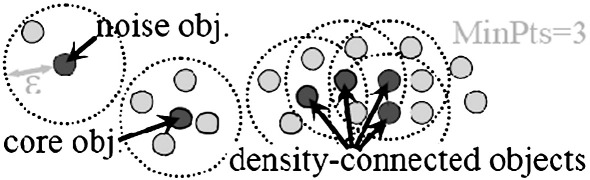
Definitions of DBSCAN.

Therefore, it can be proven that a density-based cluster can be efficiently determined by collecting all objects which are density-reachable starting from an arbitrary core object. For an illustration of the definitions of DBSCAN see Fig. 1. For *MinPts = 3*, a core object, a noise object, and a density-based cluster are displayed.

To adapt the algorithm to our setting, we redefine the core object property and direct density reachability as follows:

Modified Definitions for Voxel Clustering. Given two thresholds of Information Gain *t*_core_ and *t*_border_ and a minimum number of voxels *MinVox* we call a voxel *v*_i_ a core voxel if the IG of *v*_i_ is larger than *t*_core_ and *v*_i_ is surrounded by at least *MinVox* voxels having an IG of at least *t*_border_*.*

We allow for potentially different thresholds *t*_core_* > t*_border_ of Information Gain for core voxels and voxels at the boundaries of the clusters to require highly discriminative cluster centers and to model the natural fading of the discriminatory power in the boundary areas of the clusters. However, it is on our specific set of images not necessary to distinguish between *t*_core_ and *t*_border_ since the voxels either have a significant Information Gain value or an IG of zero. So we set *t*_core_ and *t*_border_ to the minimum IG in the data set and used *MinVox *= 6, which means that we require a core voxel to be situated in a neighborhood of highly discriminative voxels.

#### Classification

**Fig. 2:**
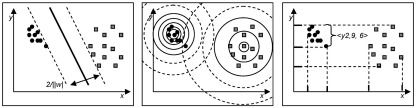
Visualizing the different classification paradigms. Left: Support Vector Machine, Center: Bayesian Classification, Right: Voting Feature Intervals.

After clustering, the selected features represent spatially coherent regions which exhibit significant differences among the groups. At this stage, classification algorithms can be applied to validate the discriminatory power of these selected clusters. Classification is a data mining (machine learning) technique used to predict group membership for data instances, which are the subjects in our application. The task of classification involves two major steps: in the so-called training phase, the classifier learns the separating information. To achieve this, some amount of instances with known class labels is required. In the test phase, the classifier predicts the class label of unlabeled instances based on the learned information. For more information on the validation of classifiers see Appendix A. Among the large variety of classifiers we chose three representative approaches with very different algorithmic paradigms. For an illustration see Fig. 2.

(1) Linear Support Vector Machine (SVM). SVM aims at constructing a hyperplane separating the training examples. Among all possible hyperplanes, SVM selects the one with the maximum margin between the training examples of both classes.
(2) Bayesian Classifier (Bayes). The fundamental idea of Bayesian classification is to model each class of the training data by a probability density function. Test objects are then assigned to most probable class.
(3) Voting Feature Intervals (VFI). Very different to SVM and Bayes, VFI is a simple entropy-based classifier. In the training phase VFI constructs class-pure intervals for each feature and each class. Classification is performed by voting.

For all classifiers, we used the implementations of the WEKA data mining toolkit available at http://www.cs.waikato.ac.nz/ml/weka/.

#### Validation

To validate the data mining framework involving the steps feature selection, clustering and classification, we used two established validation techniques: Leave-one-out cross-validation and train-and-test. Both techniques rely on the idea to learn the discriminatory patterns in a training phase on the basis of one data set which is called training data set. In the subsequent test phase, the discriminatory power of the learned pattern is evaluated using a disjoint data set, i.e. the test data. The two validation techniques differ, however, in the way the training and the test data set are composed.

##### Cross-validation

Cross-validation is an established validation scheme in the case of few training examples with respect to the dimensionality of the data (Kearns and Ron, 1997). For leave-one-out cross-validation, we divide the data set into *n* folds of size *n*-1 subjects each. In each fold, *n*-1 subjects are used for training, i.e. we perform the steps feature selection and clustering on these *n*-1 subjects and obtain a pattern of highly selective clusters. The remaining subject is used as test object, i.e. we predict the class label of this subject by applying a classifier in the feature space defined by the clusters obtained in the training phase.

##### Train-and-test

In contrast to cross-validation, which uses disjoint partitions of a single data set for validation, the train-and-test methodology employs two fully different data sets as training and test data. We apply train-and-test validation for prediction of conversion of subjects with MCI. We use HC vs. AD as training data and MCI-MCI vs. MCI-AD as test data.

##### Assessment of the classification result

To evaluate the quality of the classification result, we report three established measures: accuracy, sensitivity and specificity. The accuracy of a classifier is defined as $acc=\frac{\left|corr\right|}{n}$, whereas |*corr*| denotes the number of correctly classified subjects. The sensitivity and specificity evaluates the performance of a classifier to identify positive and negative instances, respectively, i.e. $sen=\frac{\left|TP\right|}{\left|\left(TP+FN\right)\right|}$, $spec=\frac{\left|TN\right|}{\left|\left(TN+FP\right)\right|}$, whereas |*TP*| and |*TN*| denotes the number of true positive and true negative instances, and |*FP*| and |*FN*| the number of false positives and negatives. Following a common convention, we consider a correctly identified Alzheimer's disease case, or a correctly predicted converter as a true positive.

In addition to accuracy, sensitivity and specificity, we report the 95% confidence intervals of these measures as computed by the efficient-score method (Newcombe, 1998). In particular, we applied Newcombe's fourth method, which is also commonly referred to as the Wilson procedure with continuity correction.

#### Visualization

**Fig. 3:**
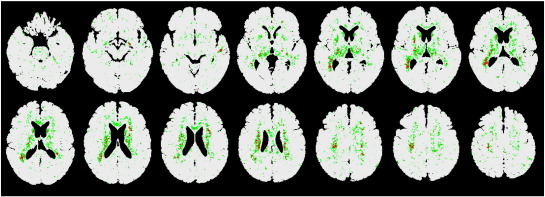
Selected features for the comparison between AD vs. HC. *z*-coordinates in Talairach space: top row of images − 45.5, − 33.5, − 26.5, − 18.5, − 13.5, − 11.5, − 5.5; bottom row: − 3.5, 0.5, 4.5, 8.5, 13.5, 15.5, 21.5.

**Fig. 4:**
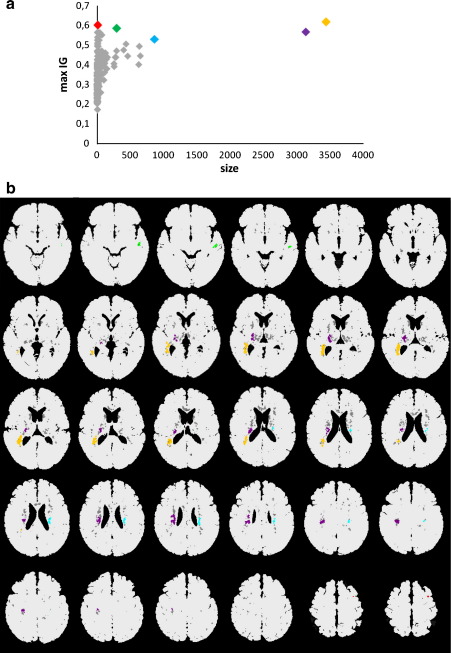
(a) Cluster size and maximum Information Gain AD vs. HC. (b) Selected features after HC vs. AD clustering. Colors: Cluster 1 red, cluster 2 green, cluster 3: blue, cluster 4 purple cluster 5 orange. Remaining clusters gray. Displayed is every second slice starting with *z* = − 31.5 to 22.5; 34.5 and 35.5.

**Fig. 5:**
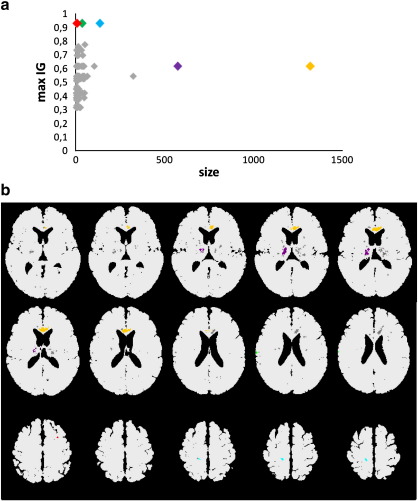
(a) Cluster size and maximum Information Gain for MCI converter vs. MCI non-converter. (b) Skyline clusters of MCI-AD vs. MCI-MCI. Colors: cluster 1: red, cluster 2: green, cluster 3: blue, cluster 4: purple, cluster 5 orange. Displayed are some representative slices containing clusters: *z*-coordinates in Talairach space: − 12.5 to 5.5 and 34.5 to 42.5 (every second slice).

We display the spatial location of the features best discriminating the classes HC and AD, and MCI-MCI and MCI-AD, respectively. For the ease of comparison, we display in Figs. 3, 4b and 5b the features which are relevant for classification in all folds. Note that this is only done to obtain one common spatial map for interpretation of the best discriminating regions and the reported classification accuracies are obtained by leave-one-out cross-validation. To facilitate interpretation, we additionally highlight the most interesting clusters in different colors in Figs. 4 and 5. We were interested in clusters which are as large as possible and exhibit an IG as high as possible. Therefore, we selected those clusters in the visualization exhibiting an outstanding combination of both criteria using the *skyline* operator which has been successfully applied in many multi-criteria decision making applications, e.g. in personalized information systems (Hristidis et al., 2001) or for the selection of web services (Skoutas et al., 2008). The skyline of a data set consists of all data objects which are not dominated by any other object in the data set with respect to any possible weighting of the studied criteria. Skylines have been studied since the 1960s and are also known as Pareto sets or admissible points (Barndorff-Nielson and Sobel, 1966). Börzsönyi et al. (2001) proposed efficient algorithms for skyline computation. To illustrate the skyline concept, imagine a user looking for hotels which are cheap and close to the beach. The hotels are represented in a database as two- dimensional feature vectors 〈*distance, price*〉. The skyline contains all offers which might be interesting to the user. By definition of dominance it can be guaranteed for all offers in the skyline that there is no better offer, i.e. there is no other hotel which is cheaper and closer to the beach. In our context, we consider clusters of voxels which are described by the features size and IG.

Anatomical location information of the clusters was obtained with the Talairach Daemon software available at http://www.talairach.org/ after MNI to Talairach coordinate transformation with the non-linear approach (Duncan et al., 2000, source-code available at http://imaging.mrc-cbu.cam.ac.uk/imaging/MniTalairach#head-b3a445e55dd349a8b2349accea51ab298c90685b).

#### SPM based voxel based analysis

A univariate voxel-based analysis using SPM 8 (Wellcome Trust Centre for Neuroimaging, London; freely available at http://www.fil.ion.ucl.ac.uk/spm/) was conducted for the group comparisons between AD vs. HC and MCI vs. HC on the basis of the deformation maps derived as described above. The default settings in SPM8 were used, with a proportional scaling and global normalization to the mean of 50. The data were spatially smoothed with a 12 mm Gaussian kernel. A significance value of *p* < 0.001 uncorrected was chosen for this exploratory analysis.

#### White matter rating

Age-related white matter rating was conducted according to a standardized procedure (Wahlund et al., 2001) by an experienced radiologist (T.M.) who was blinded to the diagnosis. Ratings were done on the basis of T2 weighted fluid-attenuated inversion recovery (FLAIR) images that were taken together with the T1 weighted MRI scans used for the classification experiments. FLAIR scans were available in a subset of 23 AD patients, 24 MCI subjects, and 6 healthy controls. The ratings of white matter hyperintensities (WMH) were done for different brain regions including the basal ganglia (including the striatum and globus pallidus), thalamus, and the internal and external capsules. In addition, ratings were done for the frontal lobe, temporal lobe, parieto-occipital lobe and the infratentorial brain area within each hemisphere.

The scores were averaged across both hemispheres subsequently. The rating scale ranges from 0 (no lesions) to a maximum score of 3 (confluent lesions) (Wahlund et al., 2001).

## Results

The mean age, MMSE and the gender distribution for AD, MCI, and HC subjects are displayed in Table 1. Nine out of 24 MCI subjects converted to AD after an average follow-up interval of 2.5 years. We applied our framework with leave-one-out cross validation on three data sets to identify highly selective brain regions for the differentiation between AD vs. HC, MCI vs. HC, and MCI converter (MCI-AD) vs. MCI non-converters (MCI-MCI). For the train-and-test validation, we used the brain regions identified in the AD vs. HC for the prediction of conversion of patients with MCI. Table 2 summarizes all experiments performed and Table 3 provides a summary of the classification results for all group comparisons, which are explained below in detail. For all classification results, also the 95% confidence interval is provided in Table 3.

### Classification of AD vs. HC

For the differentiation between AD and HC, a proportion of the voxels ranging between 97.48% and 98.04% had an Information Gain of 0, i.e. they contained no information separating the groups and were therefore excluded from further analysis.

Theoretically, combinations of these features may provide valuable information. However, due to the high dimensionality of the data, an exhaustive search for feature combinations is not applicable.

For one randomly selected fold the range of IG value among the remaining 87,416 voxels was between 0.18 and 0.69. The minimum IG of 0.18 was relatively high, indicating that the voxels either contained a good deal of valuable information to separate the classes or are completely irrelevant.

Fig. 3 displays the spatial distribution of the voxels with non-zero IG across all folds.

For one randomly selected fold clustering reduced the 87,416 selected features to 26,228. In total, 978 clusters containing at least one core object exhibiting the maximum number of 6 neighbors were obtained. The largest cluster comprised 3,445 voxels.

Fig. 4a summarizes the cluster statistics with respect to the two most important criteria: the size of the clusters and value of IG. The anatomical locations of the skyline clusters have been highlighted with the same colors in Fig. 4b. Table 4 provides a summary of the clusters including the anatomical location. Due to space limitation, only the anatomical location of the best separating regions with an IG of at least 0.3 for the large clusters 3 to 5 are included in Table 4. The clusters were centered within the medial temporal lobe including the hippocampus, parahippocampus, amygdala, adjacent basal ganglia, the right anterior cingulate gyrus extending towards the prefrontal cortex, left insula, and claustrum (Table 4).

On the basis of the selected clusters, a classification accuracy of 92% with Bayes (sensitivity: 94%, specificity: 89%), 90% with SVM (sensitivity: 97%, specificity: 78%) and 78% with VFI (sensitivity: 66%, specificity: 100%) was obtained (cf. classification task 1 in Table 3).

### Classification of MCI-AD vs. MCI-MCI

When applying feature selection on the brain images of the group of MCI with respect to conversion between 97.82% and 98.73% of the voxels have an Information Gain of 0.

For one randomly selected fold we obtained 74,680 features with IG greater than zero. The minimum occurring IG was 0.32. Clustering reduced the number of features to 10,775. The selected clusters separated converters and non-converters with high accuracy: 95.83% accuracy was obtained with SVM and VFI (sensitivity SVM: 89%, VFI 100%, specificity SVM 100%, VFI 93%), cf. task 2 in Table 3. With Bayes, an accuracy of 91.67% has been obtained (sensitivity: 78% specificity: 100%). The skyline clusters for the separation of converters and non-converters are displayed in Figs. 5a and b. The corresponding anatomical regions are provided in Table 5. In total, 276 clusters were obtained.

Using AD vs. HC as training data and MCI as test data, best results have been obtained with VFI. Conversion was predicted with an accuracy of 75% (sensitivity: 56%, specificity: 87%).

When contrasting the group of subjects with MCI against the HC for one randomly selected fold a total of 37,504 characteristic features were obtained. Clustering reduced the number of features to 2,190. On the clustered data linear SVM performed best with 97.62% in accuracy (sensitivity: 96%, specificity: 100%), cf. task 3 in Table 3. With VFI we obtained accuracy of 88.1% (sensitivity: 83%, specificity: 94%), and with Bayes an accuracy of 85.71% (sensitivity: 83%, specificity: 88%). The spatial location of the best discriminating clusters was similar to that of MCI-MCI vs. MCI-AD and therefore was not displayed.

### Parameter settings for classification

An important parameter for SVM is the complexity constant *C*. For the soft margin SVM, the parameter *C* > 0 determines the trade-off between margin maximization and training error minimization. To systematically investigate the influence of the complexity constant on the classification accuracy, we repeated the experiments with various settings for *C* in the range of log_10_ (*C*) = − 7 to + 6. The analysis was applied to 2 out of the 4 classification tasks: task 1 which involves the classification of AD vs. HC with leave-one-out cross-validation and task 4 which involves the prediction of conversion in MCI based on the model learned from AD vs. HC with train-and-test validation. We selected those two tasks for two reasons: SVM is outperformed by other classifiers in these settings (see Table 3) and the classification accuracy of SVM varies, depending upon the validation procedure applied in tasks 1 and 4.

**Fig. 6:**
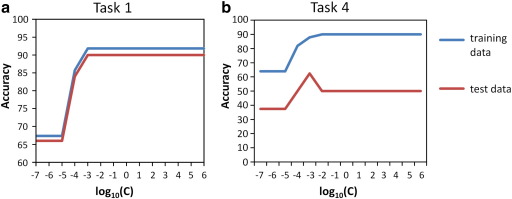
Effect of the parameter *C* on the classification accuracy of SVM in task 1 (a) and task 4 (b). For both tasks we can observe only minor influence of *C* for very small *C* ( log_10_(*C*) < − 3).

The classification result of task 1 for various settings of *C* is displayed in Fig. 6(a). Since task 1 included a leave-one-out cross-validation, the accuracy on both training and test data, respectively, were averaged among all folds. As can be seen in Fig. 6, the parameter *C* had only very minor influence on the classification accuracy. The classification accuracy for the training data (90%) and the accuracy for the test data (91.84%) were constant for a wide range of parameter settings (of log_10_ (*C*) = − 3 to 6). Only for a very small *C*, the classification accuracies for both the training and test data sets decreased numerically (log_10_ (*C*) < − 3). For log_10_ (*C*) < − 5 we observed a trend towards a statistically significant decrease in classification accuracy compared to the optimal level, i.e. the accuracy was 66% (95%CI = [52.15, 77.56]) for log_10_ (*C*) < − 5 vs. 90% (95%CI [77.41, 96.26]) for log_10_ (*C*) = − 3 to 6 when applied to the test data set.

Fig. 6b displays the result of an analogous analysis for task 4. Note that this is the most difficult classification task since the training and the test data stem from different groups of subjects. Thus, the whole data mining pipeline including feature selection, clustering and training of the SVM is applied to the training data AD vs. HC. Based on the learned model, the SVM predicts the conversion of test subjects with MCI. Fig. 6b displays the accuracy for the training data, i.e. the accuracy of the SVM applied to AD vs. HC for varying settings of the complexity constant *C*. In addition, the accuracy for the test data is displayed, i.e. the accuracy to predict conversion in MCI subjects. We can observe two different aspects from Fig. 6b. First, the training data could be well separated using a linear kernel. Since more complex kernels (like polynomial, radial basis, etc.) lead more likely to overfitting and thus lower classification accuracy when applied to the test data, their application is indicated only in the case when the training data cannot be well separated using the linear kernel. Second, visual inspection of Fig. 6b shows that the choice of the complexity parameter *C* had only very minor influence on the accuracy. Within the range of log_10_ (*C*) = − 3 to 6, the classification accuracy for the training data was constantly 90% and the accuracy for the test data was constantly 50%. Similar to the results for task 1 (see above), the classification accuracy for both the training and test data decreased only for very small *C* values. There was one single exception from this trend: For log_10_ (*C*) < − 3, we observed that the accuracy for the training data decreased from 90% to 88%, while there was a numerical increase of the accuracy from 50% to 62.5% for the test data set. However, the increase of the classification accuracy to 62.5% at *C* = 0.001 was not significantly, as it fell within the 95%CI of the classification accuracy of 50% at *C* = 1.0 (95%CI [29.65, 70.35]). Note that the parameter setting associated with a numerical increase in prediction accuracy could not be predicted on the basis of the training data, since the accuracy on training data was lower for *C* = 0.001 (88%) than for *C* = 1 (90%).

### White matter rating and SPM voxel based analysis

In order to evaluate whether the current findings may have been influenced by potential white matter damage that could lead to segmentation inaccuracies and thus bias the classification on the basis of grey matter maps, we conducted a regional rating of age-related white matter changes according to a standardized protocol (Wahlund et al., 2001). The mean scores averaged across both brain hemispheres for the basal ganglia, frontal lobe, temporal lobe, parieto-occipital lobe, and infratentorial brain area are displayed in Table 6. There were no group differences across any of the brain regions and the white matter damage was low and clinically non-significant, with, the mean rating score being below 1 for each brain area in each group.

To further validate our findings, we conducted a univariate SPM8 based group comparison of the voxel based maps. Compared to the HC group, the AD subjects showed on average grey matter reductions in the basal ganglia, medial frontal gyrus, inferior parietal lobule and precuneus for the AD vs. HC comparison (Supplementary Figure 1). MCI subjects had lower grey matter primarily within the basal ganglia and the medial and superior temporal gyrus (Supplementary Figure 2). Thus, similar brain regions affected in MCI or AD were identified in both the “classical” univariate SPM-based analysis of the deformation maps and the current classification methods, lending further support for the validity of the current findings with the novel pattern recognition method.

## Discussion

In the present study we demonstrated data mining methods to extract AD-typical patterns of brain atrophy using three different classifiers. We classified AD vs. HC, MCI-AD vs. MCI-MCI, and MCI vs. HC with excellent accuracy between 92% and 97.62% based upon leave-one-out validation. For the prediction of conversion from MCI to AD, the best predictive value was achieved with the VFI classifier reaching a predictive accuracy of about 75% validated in a train-and-test setting.

As a proof of concept we first established the AD-specific spatial pattern of atrophy using classifiers for the discrimination between AD and HC. Those brain regions that best discriminated AD from elderly HC included the medial temporal lobe, anterior cingulate gyrus extending towards the orbitofrontal cortex as well as the subcortical thalamic-basal ganglia brain areas, which were reliably identified using leave-one-out cross validation. These results are largely consistent with our previous PCA-based analysis (Teipel et al., 2007a), providing support for the convergent validity across different analysis methods. The pattern of atrophy detected in the current study agrees with findings of a range of previous independent MRI based (Meguro et al., 2001; Scahill et al., 2002) and neuropathological studies showing AD-typical predilection sites of pathological changes (Braak et al., 1997; Price et al., 1991, 2001). The current classification accuracy was high across different classifiers ranging between 78% and 92% after cross-validation.

For the classification of MCI-AD vs. MCI-MCI we obtained excellent results with a classification accuracy ranging from 91.97 to 95.83% with leave-one-out cross-validation. The skyline clusters are roughly a subset of the clusters identified for the separation of AD vs. HC, extending towards the temporal lobe including the superior temporal gyrus.

Using AD and HC as training data and MCI as test data, we achieved an accuracy of 50%–75% to predict conversion into AD. As expected, the performance of all classifiers declines in comparison to leave-one-out-cross-validation, since the test data originate from a data set that is expected to differ in the extent of pathological brain changes from the training data. These results fit with the findings of previous studies. In our previous study in the same patients, using a completely different multivariate approach based on PCA and canonical covariate analysis (Teipel et al., 2007a,b), we showed that the separation between MCI converters and non-converters was not significant, however, applying the feature vector of the AD vs. HC comparison to the MCI data resulted in an accuracy of 73% (Teipel et al., 2007a). Similarly, the group of Davatzikos (Misra et al., 2009) applied the AD vs. HC classifier (accuracy of 94%, (Fan et al., 2008)) to separate MCI converters vs. non-converters, finding a high sensitivity of 85.2% but a very low specificity of 36%, resulting in a classification accuracy of 48.5%, which is similar to the accuracy observed in our experiment (data were derived from the Fig. 6 in (Misra et al., 2009)).

In the current study, Bayes and VFI yielded superior results compared to the SVM approach. The leave-one-out experiments showed that the most selective regions for the discrimination of MCI-AD and MCI-MCI are roughly a subset of the most selective regions for AD vs. HC. Consequently, the training data contain many superfluous features, i.e. regions which are not selective to distinguish MCI-AD from MCI-MCI. VFI performs best on this difficult classification task, probably because this classifier is by design most robust with respect to superfluous information. The votes of these features approximately sum up to zero and thus have only minor effect on the classification accuracy. There is much more chance that random variations of the superfluous features cause overfitting in SVM.

Our pattern recognition technique detected structural changes within the anterior cingulate gyrus, the hippocampus and the basal ganglia in MCI converters, consistent with the previously found pattern of atrophy in MCI compared to HC (Pennanen et al., 2005). The major brain regions that separated best between HC and AD included primarily the prefrontal cortex especially the inferior and middle frontal gyri, the hippocampal region and adjacent subcortical basal ganglia, as well as more posterior brain regions within the parietal lobe. The hippocampal, frontal and parietal brain regions are well documented to be affected in AD. We detected also significant changes within the subcortical brain regions of AD. These AD-specific changes cannot be accounted for by differences in WMH, since rating of the WMH on the basis of FLAIR scans showed no group differences and the severity of WMH was in general low with the mean score always being lower than 1 across the different brain regions and diagnostic groups. The validity of the current findings is further supported by the convergent results between the univariate voxel-based analysis based on SPM and the current pattern recognition method. Furthermore, the findings on the grey matter changes within the basal ganglia are consistent with our previous results in the same patients with the PCA based approach where the component that separated best between AD and HC was strongly associated with reduced volume of subcortical brain areas including the thalamus and caudate nucleus (Teipel et al., 2007a). Thus, there is considerable overlap between different methodological approaches with regard to the detection of brain regions altered in an AD specific way. Previous independent studies have shown that considerable atrophic progression is found in subcallosal basal ganglia brain structures (Ferrarini et al., 2006; Karas et al., 2003, 2004), and was demonstrated to show one of the fastest atrophy rates within the brain of AD patients (> 15% per year, (Thompson et al., 2003)). Although there is strong evidence of atrophy within these brain regions in AD, less attention may have been spent on the basal ganglia, since the cognitive function of these brain areas is not well known. A recent study, however, that detected strong atrophy within the thalamus and putamen of AD patients showed an association with global cognitive performance and executive functions independently from hippocampus grey matter atrophy (de Jong et al., 2008). Thus, the basal ganglia structures may show pronounced volume reductions in AD.

We aimed to render our analysis especially sensitive towards the detection of subtle brain abnormalities by employing a non-linear supervised feature selection method that is less dependent upon sample-size restrained power due to cross-validation and train-and-test than previous analysis for dimensionality reduction (Davatzikos et al., 2008; Fan et al., 2008; Teipel et al., 2006). As an alternative to feature selection, dimensionality reduction can be achieved for example by principal component analysis and subsequently rated for class separation, using MANCOVA (Teipel et al., 2006, 2007a,b). However, these methods depend entirely upon data-driven transformations and thus do not reduce variability in an informed way. There exist few supervised versions of singular value decomposition (SVD) and independent component analysis (ICA, e.g. (Bair et al., 2006; Sakaguchi et al., 2002)) which consider the class labels during feature transformation. However, the results of these methods are difficult to interpret, since the amount of supervision is typically controlled by parameter settings. In contrast, the result of supervised feature selection is very intuitive because the interesting voxels are selected in the original image space. We decided to use the Information Gain as feature selection criterion, because it provides 1) a very general rating of the discriminatory power, 2) is highly efficient to compute and 3) has been successfully applied in a large variety of applications, e.g. in information retrieval (Mori, 2003), object recognition (Cooper and Miller, 1998) and bioinformatics (Yang et al., 2003; Plant et al., 2006). Correlation-based feature selection criteria, e.g. based on Pearson correlation, are closely related; however, the Information Gain is not restricted to linear correlations but captures any form of dependency between features and class labels. The applied feature selection technique can generally be used together with a wide variety of classifiers. We selected three classifiers which represent different algorithmic paradigms and therefore provide a comprehensive evaluation of the discriminatory power of the selected features.

The majority of previous studies described characteristically altered brain areas in AD or MCI on a group-level (Apostolova et al., 2007; Bozzali et al., 2006; Carlson et al., 2007; Davatzikos et al., 2001). However, the diagnostic value of group-level analysis is limited. Some studies used multivariate methods which provide the potential to draw conclusions on a single-subject level but these papers do not report validated classification results (Chen and Herskovits, 2006). Recent studies reported validated classification results for the identification of AD. Duchesne et al. (2008b) proposed to apply a support vector machine classifier based on least squares optimization on a selected volume of interest consisting of Jacobian determinants resulting from spatial normalization within the temporal lobe. In contrast, we used the whole images of the subjects as single source for feature selection and classification.

Fan et al. (2007) present an approach for identification of schizophrenia relying on deformation-based morphometry and machine learning. They achieve high classification accuracy (91.8% for female subjects and 90.8% for male subjects). This approach is conceptually similar to ours since it also applies feature selection and watershed segmentation which can be regarded as some kind of clustering, before performing classification with SVM. For each voxel, a score is computed by linearly combining the discriminatory power for classification as measured by Pearson-moment correlation with the aspect of spatial consistency which is measured by intra-class correlation. Using a similar approach, Davatzikos et al., 2008 report an accuracy of 90% for the identification of MCI in a leave-one-out validation setting. Our approach also emphasizes both aspects, the discriminatory power and the spatial coherency. However, very different definitions are applied to formalize these concepts. The discriminatory power is defined by the Information Gain, with the benefit to allow for arbitrary and not only linear correlations with the class label. Spatial coherency is achieved by density-based clustering which refines the selected features to form coherent regions. The result of watershed segmentation strongly depends on suitable selection of the thresholds which is very difficult especially in the presence of noise (Gerig et al., 1992). By the application of a modified density-based clustering technique our approach allows identifying the best discriminating brain regions without requiring any parameter settings or thresholds which are difficult to estimate. Vemuri et al. (2008, 2009a,b) showed that the predictive accuracy of SVM identified brain changes can be augmented by including other biomarkers or clinical information for the detection of AD. The highest accuracy for the AD identification was obtained when combining these imaging features with covariates, including demographic information and the apolipoprotein E genotype or cerebrospinal fluid related biomarkers.

The current study had caveats that should be taken into account for the interpretation of the results. One factor to bear in mind relates to censoring effects. Particularly at shorter follow-up intervals, censoring effects are likely to increase the number of seemingly false positives, as MCI patients with a pathologic pattern in MRI may not yet have developed clinical AD during follow-up.

It should be noted that the current study is based on a limited number of patients. In order to validate the utility of the current classifiers further application to a larger multicenter data set is necessary. In smaller samples the variability of the classification accuracy based upon the classifiers may be larger and thus less reliable (see Frost and Kallis, 2009 correspondence to Kloeppel et al., 2008, 2009). The robustness of the results and potential influence of outliers has been tested in the current study by the leave-one-out validation, but may still need further testing in larger data sets. Another caveat of the current study is that the HC group was younger when compared to the MCI group. This age difference may have influenced the results. We showed, however, previously in the same data set that age and gender were not significant predictors for the group separation based upon a PCA scores (Teipel et al., 2007a). Furthermore the focus was on distinguishing between MCI converters and non-converters who did not differ in age in the current study.

Concerning the parameter settings for classification, for SVM there are two parameter choices which may have an impact on the classification result, the choice of the kernel and the choice of the complexity constant *C*. Due to the high dimensionality of the solution space (i.e. the high number of variables) it is indicated to use a linear kernel. Other more complex kernel functions (such as polynomial, Gaussian, radial basis, etc.) are known to be subject to overfitting effects in presence of very high-dimensional spaces, i.e. good separation of the training data but deteriorated accuracy of the final classification result after validation. Therefore, more complex kernels should be used only if the training data are not well separable using the linear kernel. The results of the second tunable parameter of the SVM, the complexity constant *C*, show that the variation of this parameter within a wide range of values did not lead to any significant differences in the classification accuracy with either the leave one-out paradigm or within the training-test validation scheme. This is probably due to the fact that in all our experiments the training data are sufficiently separable by SVM using a linear kernel. In this case, the trade-off between margin maximization and training error minimization is of minor relevance in the optimization problem solved by SVM. Our results are consistent with those of LaConte et al who observed that the parameter *C* has no influence on SVM as applied to fMRI data, unless the *C* value is very small (*C* = 0.001) (LaConte et al., 2005). Vemuri et al. (2008) observed some influence of the parameter *C* on the classification result. For the classification of AD vs. HC based on MRI data only, best results with 85.8% in accuracy have been obtained using *C* = 0.01. With our method we achieved a classification accuracy of 90% for this task, independent of the selection of the parameter *C* between 0.001 and 1,000,000. Let us note that these findings are not directly comparable for several reasons: First, the study of Vemuri et al. is based on a larger collective involving 140 subjects with AD and 140 healthy controls and a different validation strategy has been applied. In our study, we applied leave-one-out cross-validation whereas Vemuri et al. applied four-fold cross validation.

In conclusion, we showed a novel approach to identify regions of high discriminatory power for the identification of AD and the prediction of conversion to AD among MCI. Our method combines data mining techniques from feature selection, clustering and classification and provides a concise visualization of the most selective regions in the original native image space. In future work we plan to apply our framework in a large multi-centre study. The study of Kloeppel et al. (2008) demonstrated the potential of SVM classification for the identification of AD in a multi-centre setting. Applying our data mining framework to a larger sample size we expect further validation of the classification results. In addition, we expect to confirm the best discriminating regions in this sample and complement them by novel findings.

## Figures and Tables

**Table 1:** Demographic variables and MMSE for the different groups.

Group	Women/men^a^	Age in years mean [SD]^b^	MMSE mean [SD]^c^
**Healthy controls**	9/9	64.8 [4.0]	29.3 [1.1]
AD patients	20/12	68.8 [8.9]	23.4 [3.0]
**MCI patients**	13/11	69.7 [8.5]	27.0 [1.8]

a Not different between groups, *χ*^2^ = 0.83 with 2 *df*, *p* = 0.66.

b One-way analysis of variance (ANOVA), *F*_2_^71^ = 2.2, *p* = 0.114, two-tailed t-test AD vs. control subjects: *t*_48_ = 1.8, *p* = 0.08, two-tailed *t*-test AD vs. MCI: *t*_54_ = 0.4, *p* = 0.69, two-tailed *t*-test MCI vs. control subjects: *t*_40_ = 2.3, *p* = 0.04.

c Significantly different between groups, Kruskal–Wallis ANOVA *χ*^2^ = 43.0, *p* < 0.001, significant difference in all pair-wise comparisons using Mann–Whitney *U* test at *p* < 0.001.

**Table 2:** Summary of classification experiments.

Task	Comparison	Validation	Training	Test
1	AD vs. HC	Leave-one-out	n.a.	n.a.
2	MCI-MCI vs. MCI-AD	Leave-one-out	n.a.	n.a.
3	MCI vs. HC	Leave-one-out	n.a.	n.a.
4	MCI-MCI vs. MCI-AD	Train-and-Test	AD vs. HC	MCI

**Table 3:** Classification results. For all classifiers and experiments, accuracy, sensitivity and specificity are provided together with the 95% confidence intervals.

Task	SVM	Bayes	VFI
*1*			
Accuracy	90% [77.41, 96.26]	92% [79.89 97.41]	78% [63.67, 88.01]
Sensitivity	96.88% [82.01, 99.84]	93.75% [77.78, 98.27]	65.63% [46.78, 80.83]
Specificity	77.78% [51.92, 92.63]	88.89% [63.93, 98.05]	100% [78.12, 100]
*2*			
Accuracy	95.83% [76.88, 99.78]	91.67% [71.53, 98.54]	95.83% [76.88, 99.78]
Sensitivity	88.89% [50.67, 99.42]	77.78% [40.19, 96.05]	100% [62.88, 100]
Specificity	100% [74.65, 100]	100% [74.65, 100]	93.33% [66.03, 99.65]
*3*			
Accuracy	97.62% [85.91, 99.88]	85.71% [70.76, 94.05]	88.1% [73.57, 95.54]
Sensitivity	95.83% [76.88, 99.78]	83.33% [61.81, 94.52]	83.33% [61.81, 94.52]
Specificity	100% [78.12, 100]	88.89% [63.93, 98.05]	94.44% [70.62, 99.71]
*4*			
Accuracy	50% [29.65, 70.35]	58.33% [28.99, 81.38]	75% [52.95, 89.4]
Sensitivity	55.56% [22.26, 84.66]	46.66% [22.22, 72.57]	55.56% [22.66, 84.66]
Specificity	46.47% [22.28,72.58]	77.77% [40.19, 96.05]	86.67% [58.39, 97.66]

**Table 4:** Clusters AD vs. HC.

Cluster-ID	Size(voxels)	Max IG	Location	Regions
5 (orange)	3,445	0.62	41.58, 28.28, − 16.98	Frontal Lobe, Inferior Frontal Gyrus, White Matter
40.59, 33.42, − 11.34	Frontal Lobe, Middle Frontal Gyrus, Gray Matter, Brodmann area 11
34.65, 16.96, − 10.52	Frontal Lobe, Extra-Nuclear, Gray Matter, Brodmann area 47
42.57, 31.32, − 14.61	Frontal Lobe, Inferior Frontal Gyrus, Gray Matter, Brodmann area 11
34.65, 24.47, 3.84	Frontal Lobe, Inferior Frontal Gyrus, Gray Matter, Brodmann area 45
41.58, 26.31, − 17.72	Frontal Lobe, Inferior Frontal Gyrus, Gray Matter, Brodmann area 47
41.58, 11.02, − 12.75	Sub-lobar, Extra-Nuclear, Gray Matter, Brodmann area 13
37.62, 22.05, − 5.73	Sub-lobar, Extra-Nuclear, Gray Matter, Brodmann area 47
34.65, 12.41, − 4.41	Sub-lobar, Insula, Gray Matter, Brodmann area 13
34.65, 17.38, − 2.13	Sub-lobar, Insula, Gray Matter, Brodmann area 47
41.58, 11.94, − 13.64	Temporal Lobe, Superior Temporal Gyrus, Gray Matter, Brodmann area 38
4 (purple)	3,135	0.57	23.76, − 4.36, − 9.45	Limbic Lobe, Parahippocampal Gyrus, Gray Matter, Amygdala
24.75, − 0.61, − 12.16	Limbic Lobe, Parahippocampal Gyrus, Gray Matter, Brodmann area 34
26.73, 2.34, − 11.47	Limbic Lobe, Subcallosal Gyrus, Gray Matter, Brodmann area 34
33.66, − 4.45, 8.05	Sub-lobar, Claustrum, Gray Matter
29.7, − 6.30, 9.99	Sub-lobar, Lentiform Nucleus, Gray Matter, Putamen
32.67, 7.31, 10.23	Sub-lobar, Claustrum, Gray Matter
32.67, 8.37, 12.02	Right Cerebrum, Sub-lobar, Insula, Gray Matter, Brodmann area 13
24.75, − 6.25, − 8.52	Sub-lobar, Lentiform Nucleus, Gray Matter, Lateral Globus Pallidus
25.74, − 4.32, − 8.62	Sub-lobar, Lentiform Nucleus, Gray Matter, Putamen
22.77, 9.92, − 15.21	Frontal Lobe, Inferior Frontal Gyrus, Gray Matter, Brodmann area 47
25.74, 4.19, − 13.24	Frontal Lobe, Subcallosal Gyrus, Gray Matter, Brodmann area 34
3 (blue)	862	0.52	− 24.75, − 0.76, 4.18	Sub-lobar, Lentiform Nucleus, Gray Matter, Putamen
− 23.76, 6.34, 10.28	Sub-lobar, Extra-Nuclear, White Matter
− 26.73, 11.09, 8.20	Sub-lobar, Claustrum, Gray Matter
− 19.8, 0.9058, − 1.30	Sub-lobar, Lentiform Nucleus, Gray Matter, Lateral Globus Pallidus
2 (green)	293	0.58	− 49.5, − 3.13, − 23.81	Temporal Lobe, Fusiform Gyrus, Gray Matter, Brodmann area 20
− 50.49, − 1.15, − 23.07	Temporal Lobe, Middle Temporal Gyrus, Gray Matter, Brodmann area 21
1 (red)	7	0.59	− 33.66, − 23.51, 34.81	Parietal Lobe, Postcentral Gyrus, Gray Matter, Brodmann area 2

**Table 5:** Clusters MCI-AD vs. MCI-MCI.

Cluster-ID	Size (voxels)	Max IG	Location	Regions
5 (orange)	1,320	0.61	− 1.98, 47.87, − 5.59	Anterior Lobe, Culmen, Gray Matter
4 (violet)	573	0.62	15.84, − 0.27, − 5.45	Sub-lobar, Lentiform Nucleus, Gray Matter, Medial Globus Pallidus
15.84, 1.66, − 5.55	Sub-lobar, Lentiform Nucleus, Gray Matter, Lateral Globus Pallidus
14.85, − 7.85, − 1.71	Sub-lobar, Extra-Nuclear, White Matter
19.80, 3.69, − 3.97	Sub-lobar, Lentiform Nucleus, Gray Matter, Putamen
3 (blue)	135	0.93	16.83, 14.50, 37.50	Frontal Lobe, Cingulate Gyrus, Gray Matter, Brodmann area 32
18.81, 15.33, 34.67	Frontal Lobe, Cingulate Gyrus, White Matter
20.79, 16.34, 35.57	Frontal Lobe, Sub-Gyral, White Matter
18.81, 16.26, 33.73	Limbic Lobe, Cingulate Gyrus, White Matter
14.85, 19.39, 38.18	Limbic Lobe, Sub-Gyral, White Matter
2 (green)	35	0.93	67.32, − 0.67, 6.02	Temporal Lobe, Superior Temporal Gyrus
1 (red)	7	0.93	− 27.72, − 23.65, 32.04	Frontal Lobe, Sub-Gyral, White Matter

**Table 6:** Mean rating scores of age related white matter changes and standard deviation (in brackets) for each group and different brain regions.

Group	Brain region				
Basal ganglia	Infratentorial area	Frontal lobe	Temporal lobe	Parieto-occipital Lobe
AD	< 0.1 (< 0.1)	0 (0)	0.5 (0.6)	0.2 (0.4)	0.5 (0.5)
MCI	< 0.1 (0.1)	0 (0)	0.6 (0.7)	0.2 (0.4)	0.5 (0.7)
HC	0 (0)	0 (0)	0.3 (0.5)	0 (0)	0.2 (0.4)

## Appendix A

### Linear Support Vector Machine (SVM) (Platt, 1998)

As a function-based classifier, the Linear Support Vector Machine aims at constructing a hyperplane separating the training examples. Originally, the Support Vector Machine has been designed for two class problems and therefore we restrict ourselves to this case. For data sets of dimensionality *d* there are often many possible separating hyperplanes of dimensionality *d*-1, especially for high dimensional data. To predict the class of unknown objects with highest possible accuracy and to avoid over fitting, among all separating hyperplanes the one maximizing the margin between the training examples of both classes needs to be determined. Formally, a separating hyperplane can be defined by:

$$\langle w,s_{i}\rangle +b=0$$

The class label of each subject *s*_*i*_ is determined by the signum function of the separating hyperplane. The location of the hyperplane is described by the vector *w* which is perpendicular to the plane and the bias *b* which specifies its shift from the origin of the coordinate system. To find the hyperplane providing the largest margin between both classes, only the closest instances to the plane at both sides are of interest, the so-called support vectors. If the classes are linearly separable, the maximum margin hyperplane is determined by parallel hyperplanes passing through the support vectors with maximum distance from each other (cf. Fig. 2). Since the distance between those hyperplanes equals 2/‖*w*‖, selecting the largest margin hyperplane means minimizing ‖*w*‖ subject to the constraint of a correct classification of the training examples. This so-called primal optimization problem can be efficiently solved by dynamic programming. The optimization problem can be rewritten by expressing *w* in terms of scalar products of the support vectors. In this dual form kernel functions can be applied if the data is not linearly separable in the original space. An extension is the soft margin support vector machine which allows misclassified instances within the margin to counteract over fitting. For soft margin classification, there is a trade-off between minimizing ‖*w*‖ and the number of misclassified instances, i.e. between margin maximization and training error minimization. This trade-off is controlled by a parameter, the so-called complexity constant *C*.

### Bayesian Classifier (Bayes) (John and Langley 1995)

Bayesian classification relies on the assumption that each feature (in our application each voxel) follows a probability density function, in most approaches a Gaussian distribution is assumed. Each class can thus be characterized by a potentially different mixture model of *d* probability density functions. Classification is performed by assigning the object to the most probable class, i.e.

$$\underset{c_{i}\in C}{s_{i}.c=\arg\max}P\left(v_{1},\ldots ,v_{d}|c_{i}\right)\cdot P\left(c_{i}\right).$$

The probability of each class *P(c_i_)* can be interfered easily from the training data. However, it is in most applications impossible to estimate the conditional probability *P(v_1_,…, v_d_|c_i_)*, since for each class several instances *V = {v_1_,…,v_d_}* would be needed. Therefore, the Naïve Bayesian classifier relies on the simplifying assumption that the single features are independent of each other, i.e. $P\left(v_{1},...v_{d}|c_{i}\right)=\prod_{j=1}^{d}P\left(v_{j}|c_{i}\right).$ The decision rule is simplified to

$$s_{i}.c\underset{c_{i}\in C}{\arg\max}P\left(c_{i}\right)\cdot \prod_{j=i}^{d}P\left(v_{j}|c_{i}\right).$$

See Fig. 2 for an example of two classes which are modeled by Gaussian distributions. In spite of the fact that the assumption of independence does not hold in many applications including MRI data (neighboring voxels are usually highly correlated), Naïve Bayesian classifiers often show good predictive performance. The Bayesian classifier used in this study extends Naïve Bayesian classification by the application of Parzen Windows with Gaussian Kernel to estimate the distributions of continuous attributes (John and Langley, 1995). The derived distributions of the features are thus not restricted to be Gaussian which has been demonstrated to improve the performance of Bayesian classification on many real-world data sets.

### Classification by voting feature intervals (VFI) (Demiroz and Guvenir, 1997)

This simple entropy-based classifier constructs intervals for each class and each feature and records class counts. Classification is performed by voting. During the training phase the intervals, also called concepts are constructed as follows:

For each of the *d* features (i.e. voxels *v_1_,…,v_d_*) and for each of the *k* classes *c_1_,…,c_k_* the maximum and the minimum value of *v*_i_ in class *c*_*j*_ is determined. The list of *2k* end points is sorted and each pair of consecutive points represents an interval. Each interval can be represented as a vector 〈lower, count*_1_, …* count_*k*_〉 where *lower* denotes the lower bound and count*_1_, …,* count_*k*_ the number of subjects of each class having an intensity value of voxel *v*_i_ within the interval. An example interval with the starting point *y2* containing 9 subjects of one class and 6 subjects of the other class is visualized in Fig. 2.

To classify a subject *s*, for all d voxels the intervals in which they fall are determined. For each interval *I* and each class *c*_*i*_ a vote is computed as follows:

$${\text{vote}}_{I,c_{i},v_{j}}=\frac{\text{IntervalClassCount}\left(c_{i}\right)}{\left|c_{i}\right|},$$

where IntervalClassCount(*c*_*i*_) denotes the number of subjects of class *c*_*i*_ which have an intensity of voxel *v*_*j*_ within the interval *I.* The votes are scaled between 0 and 1 and the final class prediction is computed by summing up all votes.

## Appendix A Supplementary data

Supplementary Table 1 Univariate ANOVA with diagnosis (AD, MCI, controls) as independent factor and jacobian determinant maps. Proportional scaling to global mean 50, smoothing 12 mm FWHM.

Supplementary Figure 1 Reductions of brain in AD patients vs. controls projected onto the template brain in MNI standard space. The sections go from Talairach–Tournoux coordinate *z* = − 13 to *z* = 47, sections are spaced 4 mm apart. The brain is viewed from superior to inferior, the right side of the image corresponds to the right side of the brain.

Supplementary Figure 2 Reductions of brain in MCI patients vs. controls projected onto the template brain in MNI standard space. The sections go from Talairach–Tournoux coordinate *z* = − 13 to *z* = 47, sections are spaced 4 mm apart. The brain is viewed from superior to inferior, the right side of the image corresponds to the right side of the brain.
