# Supplementary material for: Automated detection of brain atrophy patterns based on MRI for the prediction of Alzheimer's disease
Source: Neuroimage. 2010 Mar;50(1):162–74. doi: 10.1016/j.neuroimage.2009.11.046 (PMC2838472; doi:10.1016/j.neuroimage.2009.11.046)
Supplement: Supplementary Table 1 — Univariate ANOVA with diagnosis (AD, MCI, controls) as independent factor and jacobian determinant maps. Proportional scaling to global mean 50, smoothing 12 mm FWHM. [file mmc1.doc]

Univariate ANOVA with diagnosis (AD, MCI, controls) as independent factor and jacobian determinant maps. Proportional scaling to global mean 50, smoothing 12 mm FWHM.

**Table 1: Univariate effect - brain reductions in AD vs. controls**

|  |  |  | **Coordinates (mm)** | | |  |
| --- | --- | --- | --- | --- | --- | --- |
| **Region** | **Side** | **BA** | **x** | **y** | **z** | **T_71_** |
| **Frontal lobe white matter** | Right |  | 31 | -18 | 29 | 6.12 |
| **Frontal lobe white matter** | Right |  | 31 | 6 | 35 | 5.65 |
| **Frontal lobe white matter** | Left |  | -26 | -16 | 27 | 5.55 |
| **Inferior parietal lobule white matter** | Left |  | -50 | -47 | 38 | 4.17 |
| **Precuneus white matter** | Right |  | 8 | -56 | 48 | 3.81 |
| **Precuneus** | Left | 7 | -8 | -49 | 44 | 3.70 |
| **Medial frontal gyrus white matter** | Right |  | 1 | 46 | 41 | 3.56 |
| **Medial frontal gyrus** | Left | 10 | -5 | 49 | 11 | 3.54 |
| **Medial frontal gyrus white matter** | Right |  | 37 | 14 | 54 | 3.49 |
| **Corpus callosum truncus** | Right |  | 10 | -6 | 23 | 3.34 |

**Table 2: Univariate effect - brain reductions in MCI vs. controls**

|  |  |  | **Coordinates (mm)** | | |  |
| --- | --- | --- | --- | --- | --- | --- |
| **Region** | **Side** | **BA** | **x** | **y** | **z** | **T_71_** |
| **Lentiform nucleus** | Left |  | -23 | 2 | 1 | 5.31 |
| **Internal capsule WM** | Right |  | 26 | -17 | 23 | 5.09 |
| **Lentiform nucleus** | Right |  | 26 | -14 | 8 | 4.83 |
| **Cerebellum** | Right |  | 24 | -33 | -19 | 5.11 |
| **Medial temporal gyrus WM** | Right |  | 37 | -49 | 9 | 4.85 |
| **Caudate Nucleus** | Right |  | 11 | 23 | 10 | 3.58 |
| **Superior temporal gyrus GM** | Right | 22 | 62 | -50 | 16 | 3.57 |
| **Superior temporal gyrus WM** | Right |  | 39 | -57 | 29 | 3.51 |
