# Supplementary material for: Automated detection of brain atrophy patterns based on MRI for the prediction of Alzheimer's disease
Source: Neuroimage. 2010 Mar;50(1):162–74. doi: 10.1016/j.neuroimage.2009.11.046 (PMC2838472; doi:10.1016/j.neuroimage.2009.11.046)

**Supplementary Figure 1: Reductions of brain in AD patients vs. controls projected onto the template brain in MNI standard space. The sections go from Talairaich–Tournoux coordinate z = -13 to z= 47, sections are spaced 4 mm apart. The brain is viewed from superior to inferior, the right side of the image corresponds to the right side of the brain.**

**
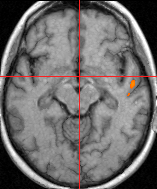

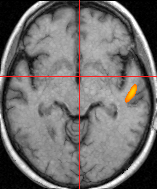

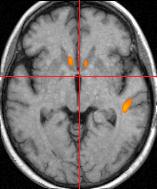

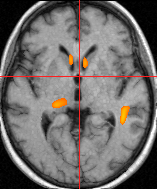

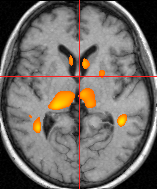

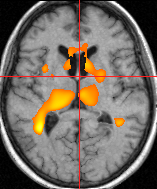

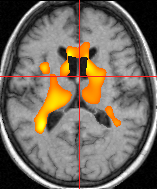

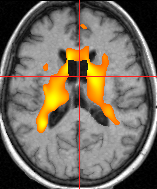

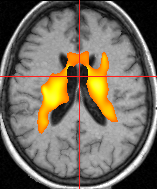

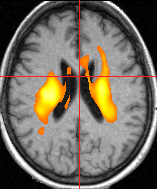

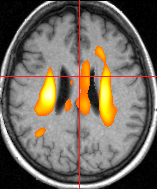

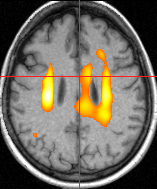

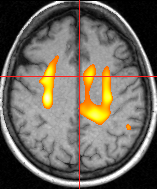

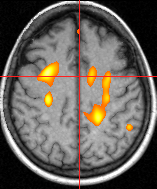

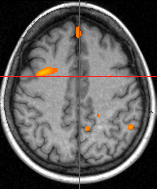

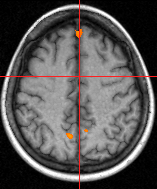
**

**Supplementary figure 2: Reductions of brain in MCI patients vs. controls projected onto the template brain in MNI standard space. The sections go from Talairaich–Tournoux coordinate z = -13 to z= 47, sections are spaced 4 mm apart. The brain is viewed from superior to inferior, the right side of the image corresponds to the right side of the brain.**


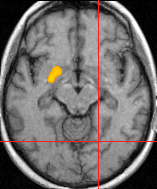

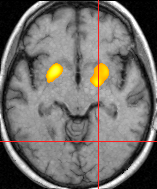

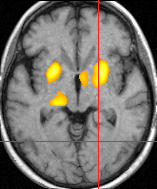

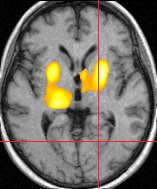

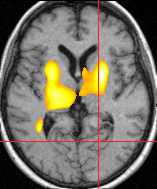

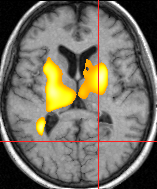

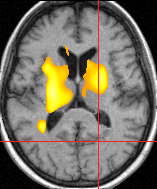

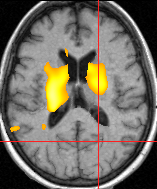

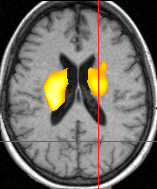

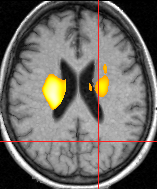

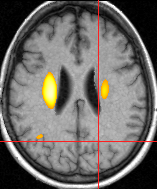

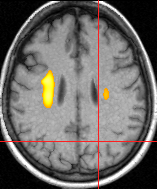

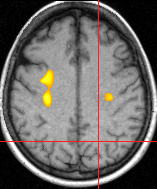

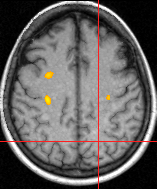

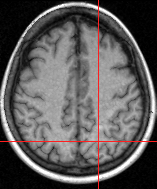

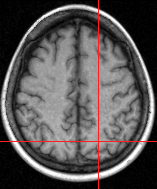

Supplement: Supplementary Figure 1 — Reductions of brain in AD patients vs. controls projected onto the template brain in MNI standard space. The sections go from Talairach–Tournoux coordinate z = − 13 to z = 47, sections are spaced 4 mm apart. The brain is viewed from superior to inferior, the right side of the image corresponds to the right side of the brain. [file mmc2.doc]
